# Impact of preformed T-cell alloreactivity by means of donor-specific and panel of reactive T cells (PRT) ELISPOT in kidney transplantation

**DOI:** 10.1371/journal.pone.0200696

**Published:** 2018-07-30

**Authors:** Ilaria Gandolfini, Elena Crespo, Mukta Baweja, Marta Jarque, Chiara Donadei, Sergio Luque, Núria Montero, Anna Allesina, Laura Perin, Umberto Maggiore, Paolo Cravedi, Oriol Bestard

**Affiliations:** 1 Department of Medicine, Translational Transplant Research Center, Icahn School of Medicine at Mount Sinai, New York, NY, United States of America; 2 Kidney and Kidney-Pancreas Transplant Unit (Department of Nephrology), Parma University Hospital, Parma, Italy; 3 Experimental Nephrology Laboratory, IDIBELL, Barcelona University, Barcelona, Spain; 4 Kidney Transplant Unit, Bellvitge University Hospital, IDIBELL, Barcelona University, Barcelona, Spain; 5 GOFARR Laboratory, Saban Research Institute, Division of Urology, Children's Hospital Los Angeles, Los Angeles, California, United States of America; 6 Department of Urology, Keck School of Medicine, University of Southern California, Los Angeles, California, United States of America; INSERM, FRANCE

## Abstract

Donor-specific (d-sp) interferon gamma enzyme-linked immunosorbent spot (d-sp ELISPOT) and Panel of reactive T-cell (PRT) ELISPOT assays have been developed to detect alloreactive memory T (Tmem) cells in order to estimate the risk of acute rejection after kidney transplantation. Adding IL15 to the PRT assay (PRT+IL15) may uncover the presence of pathogenic alloreactive CD28^-^Tmem. Face-to-face comparisons of these assays have not been done yet. We performed pre-transplant d-sp ELISPOT and PRT assays (±IL15, against six B-cell lines) in 168 consecutive kidney transplant recipients and evaluated the multivariable-adjusted associations with biopsy-proven acute rejection (BPAR), *de novo* donor-specific antibodies (DSA), and eGFR decline over a 48-month follow-up period. D-sp ELISPOT was positive in 81 (48%) subjects, while 71 (42%) and 81 (48%) subjects displayed positive PRT and PRT+IL15, respectively. Their median [interquartile range] numerical test result was 23 [6–65], 18 [8–37], and 26 [10–45] spots/3x10^5^ PBMCs, respectively. The number of PRT spots were weakly correlated with those of d-sp ELISPOT, but highly correlated with PRT+IL15 (rho = 0.96, P<0.001). d-sp ELISPOT, but not PRT (±IL15) was independently associated with BPAR (adjusted Odds Ratio of BPAR associated with d-sp ELISPOT positivity: 4.20 [95%CI: 1.06 to 21.73; P = 0.041]). Unlike d-sp ELISPOT, median PRT and PRT+IL15 were independently associated with higher Δ3-48month eGFR decline post-transplantation (for both assays, about -3mL/min/1.73m2 per one standard deviation unit increase in the spot number). Pre-transplant T-cell immune-monitoring using d-sp ELISPOT and PRT assays identifies kidney transplant candidates at high risk of BPAR and worse kidney allograft progression.

## Introduction

Currently, immunosuppressive therapy in kidney transplant patients is largely chosen on the basis of center-specific protocols and is empirically guided by nonspecific clinical parameters including serum creatinine, circulating drug levels, and kidney biopsies [1–4]. As a result, there are some patients receiving too little immunosuppression and others unnecessarily exposed to the toxicities of inadequately high doses of immunosuppressive drugs [5,6]. Therefore, tools to monitor alloimmune response in a non-invasive and specific manner are urgently needed to tailor immunosuppression according to the individual-patient immunological risk [7–11].

The notion that alloreactive memory T cells (Tmem) are crucial mediators of allograft rejection led to the development of the cytokine enzyme-linked immunospot (ELISPOT) assay which is able to quantify circulating alloreactive Tmem at the single cell level [12,13]. Initial studies have shown that pre-transplantation d-sp ELISPOT correlates with biopsy-proven acute rejection (BPAR) after kidney transplantation [14,15] and could be used to tailor immunosuppression [16–19]. In 2015, a large prospective-cohort study of 176 kidney transplant patients surprisingly failed to observe a relationship between positive pre-transplant d-sp ELISPOT and BPAR, but detected a relationship between and lower one-year graft function in patients not receiving thymoglobulin induction. This relationship was absent in patients induced with thymoglobulin, suggesting that thymoglobulin diminishes the risk of graft injury in patients with a positive d-sp ELISPOT before transplant [20].

A major drawback of the d-sp ELISPOT assay is that it requires donor cells and over 24 hours to be performed, making it impractical in cadaveric kidney recipients [21]. To address this issue, a T-cell reactivity index, or panel of reactive T-cells (PRT) has been proposed utilizing the ELISPOT responses to common HLA antigens from a pool of donors reflective of general organ donor pool. Similar to the panel-reactive antibody test for identifying individuals with elevated levels of anti-HLA antibodies, the PRT may identify patients at risk for post-transplantation cellular-mediated graft injury [21]. In a small study of 30 kidney transplant patients, six of the seven (86%) patients with acute rejection were PRT-positive whereas only one had low PRT before transplantation [22]. Other small, retrospective studies reported an association between positive pre-transplant PRT and increased risk of acute rejection [23]. However, the utility of PRT in predicting graft outcomes has not yet been investigated in larger cohorts of kidney transplant patients. Additionally, a comparison of the performance of the d-sp ELISPOT assay and the PRT assay has not been evaluated yet.

Emerging data identified circulating CD28^-^ T cells as crucial population for both allograft tolerance and rejection [24–26]. These cells do not proliferate nor produce IFN-γ in regular mixed lymphocyte reaction assays, but can be unraveled by adding IL-15 to the assay [27,28]. In vivo, IL-15 is produced by renal epithelial cells and promotes the recruitment and activation of alloreactive CD28^-^ T cells. Therefore, quantifying these cells pre-transplant by adding IL15 to standard PRT may be important in stratifying the risk of BPAR [27].

In this large retrospective-cohort study, we performed pre-transplant d-sp ELISPOT and PRT (±IL15) in 168 consecutive kidney transplant recipients and evaluated their independent relationship with the development of BPAR and other transplant outcomes including *de novo* DSA, graft function and graft failure.

## Materials and methods

### Patients and interventions

This retrospective-cohort study included all consecutive adult patients (≥18 years) who received a kidney transplant from living or deceased donors at Bellvitge University Hospital, Barcelona, Spain, from 2011 to 2013 who had donor and recipient blood or spleen samples available. Exclusion criteria included multiple organ transplant recipients, ABO incompatible transplants, and positive complement-dependent cytotoxicity cross-match. The study was approved by Institutional Review Board (IRB) (HUB PR228/13) of the Bellvitge University Hospital and all eligible patients provided written informed consent for study participation. None of the transplant donors were from a vulnerable population and all donors or next of kin provided written informed consent that was freely given.

Estimated GFR (CKD-EPI) [29] and 24h-proteinuria were assessed at 3, 6, 12, 24, 36 and 48 months post-transplantation. All patients underwent graft biopsy in the case of clinical dysfunction. All biopsy samples were scored following the international Banff 2013 classification criteria [30].

Delayed graft function (DGF) was defined as the need for dialysis during the first week after transplant. Patients were followed-up until October 31^st^ 2016 or graft failure (dialysis or death with a functioning graft).

Immunosuppressive therapy consisted of induction with thymoglobulin (total dose: 4.5 mg/kg over 5 days) or basiliximab (20 mg on days 0 and 4). Maintenance immunosuppressive treatment consisted of tacrolimus or cyclosporine, mycophenolate mofetil (MMF) and steroids. Steroid withdrawal was undertaken at 3 months after transplant based on clinical criteria in those patients with stable renal function and no previous BPAR episodes.

Donor-specific ELISPOT and PRT results were unavailable to the clinicians in charge of the patients and therefore had no influence on the choice of immunosuppression and clinical management of the transplant patients.

### Donor-specific ELISPOT assay

Donor-specific ELISPOT assay was performed as previously described [16,31]. In brief, recipient peripheral blood mononuclear cells (PBMCs) (harvested on the day of transplant before the administration of any immunosuppressive drug) were isolated by Ficoll gradient centrifugation. Donor cells were obtained from donor spleens or PBMCs in deceased and living donors, respectively. Donor and recipient cells were frozen in liquid nitrogen and defrosted on the day of the ELISPOT or PRT assays. Deceased-donor splenocytes were CD2-depleted and living-donor PBMCs were CD3-depleted. Recipient PBMCs (3x10^5^ cells/well) were tested in triplicate wells with respective donor cells (3x10^5^ cells/well) in 96-well plates. Anti-third party cells (full mismatch A, B and DR splenocytes) were also used as stimulators and evaluated in triplicate wells. PBMCs plus medium alone served as a negative control and phytohemagglutinin (PHA) stimulation was used as a positive control.

### PRT assay

The lowest number of stimulator B cells and PBMCs able to provide results highly correlated (**S1 Fig**; R2: 0.94; P<0.0001) with the ones obtained with standard technique [22,23] was 100.000 responder PBMCs against 60.000 *in vitro* expanded (not EBV transformed) allogeneic B-cell stimulators. Therefore, we used these numbers of cells for all our experiments in a 384-well plate. Each responder was tested in duplicate against a panel of six previously frozen B-cell stimulators (HLA typing provided in **S1 Table**) with or without the addition of IL15 (1 ng/mL, Biolegend). PBMCs plus medium alone or IL15 served as negative controls for PRT and PRT-IL15, while PHAserved as positive controls.

We optimized PRT IL-15 assay by testing increasing concentrations of IL-15: 0.5, 1, 3, 5, 7, 10, 50, and 100 ng/mL. We found that 1 ng/mL of IL-15 was able to increase the number of recipient PBMCs reactivity against B-cells without significantly increasing the number of spot in PBMCs not exposed to B cells and only to medium alone (data not shown).

B cell lines were obtained from Dr. Heeger (Icahn School of Medicine at Mount Sinai, New York, US) [22]. Briefly, B cells were isolated and expanded from a panel of from 6 distinct donor spleen cells or PBMC. B cells have been expanded in vitro by culturing them with cytokines (IL-2 and IL-4) and CD40L transfected fibroblasts. In order to confirm that B cell lines represented donor HLA repertoires in our cohort of patients, the expression of common HLA A, B and DR alleles was studied in this cohort regarding B cell lines HLA typing. As shown in **S2 Fig**, B cell panel HLA alleles represented 61.95%, 59.27% and 78.86% of expression over all HLA A, HLA B and HLA DR alleles expressed in our donor cohort, respectively. Importantly, besides covering a great proportion of our donor HLA repertoire, the HLA type of the B cell lines represented the most frequent alleles of our donor cohort population.

### Spot quantification

The spots for d-sp ELISPOT were quantified using the AID® ELISPOT reader 4th generation (Autoimmun Diagnostika, Strassberg, Germany) and for PRT using the ImmunospotS4 Core Analyzer (CTL, Shaker Heights, OH, USA) by two independent researchers and averaged. We determined mean numbers of d-sp ELISPOTs per 3x10^5^ responder PBMCs from triplicate wells. Spots detected in control wells without stimulators were subtracted from the total number of spots.

To determine d-sp ELISPOT positivity, we used the previously published threshold of ≥25 IFN-γ spots/3x10^5^ PBMC^14^. This choice was supported by an analysis aimed at identifying the best cut-off point (data not shown), that compared the Akaike information criterion between regression models for BPAR which differed for the selected d-sp ELISPOT positivity cutpoint.

We analyzed PRT and PRT +IL15 as continuous variables represented by the median number of spots/3x10^5^ PBMCs against the 6 B-cell lines. We defined PRT positivity against a B cell line as ≥40 spots/3x10^5^ PBMCs, and ≥50 spots/3x10^5^ for PRT +IL15 (cut-offs that were the approximate lower bound of the upper quintile of median PRT and PRT+IL15, respectively). Also, patients were classified as belonging to the category of “positive” PRT when PRT ELISPOTs were positive against at least one of the six B-cell lines, or to the “negative” PRT category when PRT ELISPOTs were negative against all the six B-cell lines.

### Circulating anti-HLA antibodies

Screening for circulating anti-HLA class I and II alloantibodies was done in all patients prior to transplantation and in a subset of 117 patients (69%) at least once after transplantation according to serum availability, using single-antigen flow bead assays on a Luminex platform (Lifecodes, a division of Immucor, Stamford, CT). All beads showing a normalized mean fluorescence intensity of >1500 MFI were considered positive if (mean fluorescence intensity/[mean fluorescence intensity lowest bead]) > 5.

### Statistical analyses

All analyses were performed using the statistical package Stata Statistical Software package, Release 15.0. (StataCorp, College Station, TX). A two-tailed p-value less than 0.05 was regarded as statistically significant.

Estimates were expressed as differences between positive vs. negative d-sp ELISPOT, differences between positive vs negative PRT (and PRT+IL15), or as changes per one unit standard deviation of the continuous variables of d-sp ELISPOTs, median PRT, and median PRT+IL15, which were approximately 50, 25, and 30 IFN-γ spots/3x10^5^ PBMCs for d-sp ELISPOT, PRT and PRT+IL15, respectively. Linearity of the continuous variables was tested using fractional polynomials.

We estimated the association between baseline recipients’ characteristics and the mean number of IFN-γ spots of d-sp ELISPOT, PRT and PRT+IL15 using gamma regression via generalized linear models with robust standard errors, due to the non-normal distribution with long right tails of the dependent variables.

We used the Kaplan–Meier method to estimate the crude probability of uncensored and death-censored graft survival and Cox regression models to examine the multivariable-adjusted relationship between d-sp ELISPOT, PRT, PRT+IL15 and graft failure. Logistic regression models, with the statistical inference based on the likelihood ratio test, were used to estimate multivariable-adjusted odds ratios of BPAR and de novo DSA associated with d-sp ELISPOT and PRT (and PRT+IL15). Because of the virtual absence of BPAR-outcomes in patients receiving thymoglobulin induction, all the analyses were repeated in the subset of patients not receiving thymoglobulin induction.

For the analysis of 48-months longitudinal changes of eGFR and of Log(proteinuria) from baseline, set at 3 months post-transplantation, we fitted repeated measures linear mixed models using restricted maximum likelihood to take into account of the presence of unbalanced data (i.e. not all patient had the eGFR measured at each time point). All the reported hypothesis tests for the fixed effects were based on a small-sample adjustment [32]. We checked normality distribution assumption by inspecting histograms and standardized normal probability plot of residuals, and homogeneity of variance assumption by inspecting residuals-vs-fitted plots. We verified model fitting by inspecting observed-vs-fitted-values plots, and observed-vs-fitted-individual-eGFR-trajectories plots.

All multivariable-adjusted regression models included, whenever applicable, the following characteristics: baseline eGFR, baseline 24h-proteinuria, recipient and donor age, living (vs deceased) donor, cold ischemia time, thymoglobulin induction (indicator variable 1 if yes, 0 if otherwise), re-transplantation, pre-transplant HLA antibodies (indicator variable 1 if cPRA >5%, 0 if otherwise), HLA A/B and HLA DR mismatch, glomerulonephritis as primary renal disease (indicator variable 1 if yes, 0 if otherwise), dialysis vintage, and prednisone withdrawal.

We used the *margins* Stata command to calculate crude and adjusted means, crude and adjusted effects and 95% confidence intervals, as predicted by the previously fitted regression model.

## Results

### Patients

The study included 168 patients who were followed-up for a median (interquartile range) period of 45 (37–61) months. Patients were mainly Caucasian and recipients of cadaveric donors (**Table 1**). Only a minority of recipients were at increased immunological risk either because of re-transplantation (13%) or because of pre-formed HLA circulating antibodies (12%). Most patients received induction therapy, which was based on thymoglobulin or basiliximab in 41 (24%) and 109 (65%) cases, respectively. Maintenance immunosuppression was based on calcineurin inhibitors, MMF, and steroids. Steroid withdrawal was undertaken in 57% of the subjects at 3 months after transplant. Donor-specific ELISPOT and PRT (±IL15) data were available for all the patients.

**Table 1:** Demographic and clinical characteristics of the study population.

Number of subjects		168
**Living Donor**	*%*	35 (20.8)
**Donor Age**	*yrs*	57.7 (16.0)
**Recipients Age**	*yrs*	56.2 (13.4)
**Males**	*%*	108 (64.3)
**Caucasian Race**	*%*	159 (94.6)
**Dialysis vintage**	*months*	30.6 (6.9–44.9)
**Glomerular ESRD**	*%*	34 (20.2)
**Vascular ESRD**	*%*	16 (9.5)
**Tubulo-interstitial ESRD**	*%*	20 (11.9)
**Diabetes ESRD**	*%*	20 (11.9)
**ADPKD ESRD**	*%*	28.(16.7)
**Others and unknown**	*%*	50 (29.8)
**CIT (deceased donor)**	*hours*	19.1 (4.3)
**HLA-I mm**	*number*	2.7 (2–3)
**HLA-II mm**	*number*	1.0 (1–1)
**Re-transplant**	*%*	22 (13.1)
**cPRA>5%**	*%*	12 (7.1)
**D+/R+ CMV status**	*%*	127 (75.6)
**D+/R- CMV status**	*%*	10 (5.9)
**D-/R+- CMV status**	*%*	24 (14.3)
**D-/R- CMV status**	*%*	7 (4.2)
**Thymoglobulin**	*%*	41 (24.4)
**Basiliximab**	*%*	109 (64.9)
**No induction**	*%*	18 (10.7)
**Tacrolimus**	*%*	150 (89.3)
**Cyclosporine**	*%*	18 (10.7)
**Steroid withdrawal**	*%*	96 (57.1)
**Median d-sp ELISPOT**	*spots/3x10*^*5*^*PBMC*	23.0 (6–65)
**Median PRT**	*spots/3x10*^*5*^*PBMC*	18.1 (7.9–36.8)
**Median IL-15 PRT**	*spots/3x10*^*5*^*PBMC*	26.4 (10.4–45.4)

Continuous variables are reported as mean (standard deviation) or median (interquartile range), categorical variables as number (percentage). D/R donor/recipient positive (+) or negative (-) CMV serum status; ESRD, End Stage Renal Disease; PRT, Panel Reactive T-cell ELISPOT; d-sp ELISPOT, Donor-specific ELISPOT; cPRA, calculated Panel Reactive Antibody; DSA, Donor-specific antibodies; eGFR, estimated GFR (CKD-EPI formula). Median PRT and median PRT+IL15, median number of spots against the six B-cell lines.

### Donor-specific ELISPOT and PRT

Pre-transplant donor-specific and PRT ELISPOTs were not associated with main baseline clinical, immunological or demographic characteristics (data not shown). Donor-specific ELISPOT was positive in 81 (48%) patients, while 71 (42%) and 81 (48%) patients had a positive PRT and a PRT+IL15 against at least one of the six B-cell lines, respectively. The number of spots was of the same order of magnitude across d-sp ELISPOTs, PRT, and PRT+IL15 (50° percentile: 23, 18 and 26 IFN-γ spots/3x10^5^ PBMCs, respectively), but d-sp ELISPOT had the largest variability among the three assays as judged by its interquartile range (**Table 1**).

**Fig 1:**
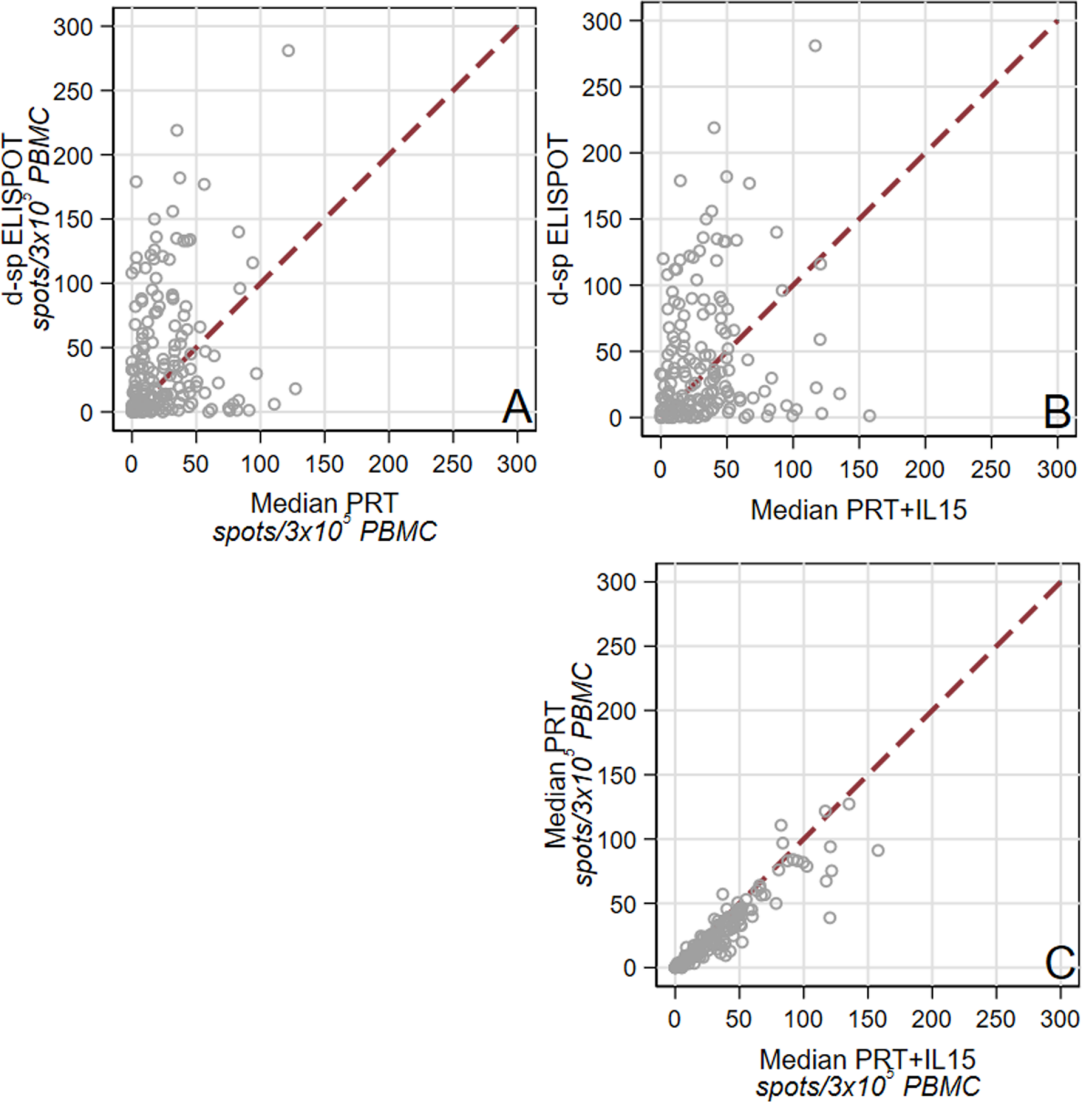
**Correlation between d-sp ELISPOT (as number of spots) and median (*i*.*e*., median number of spots against the six B cell lines) PRT (A) and PRT+IL-15 (B)** (rho = 0.18, P = 0.021 and rho = 0.19, P = 0.016, respectively). **Correlation between median PRT and median PRT IL-15** (rho = 0.96, P<0.001) (**C**).

**Fig 2:**
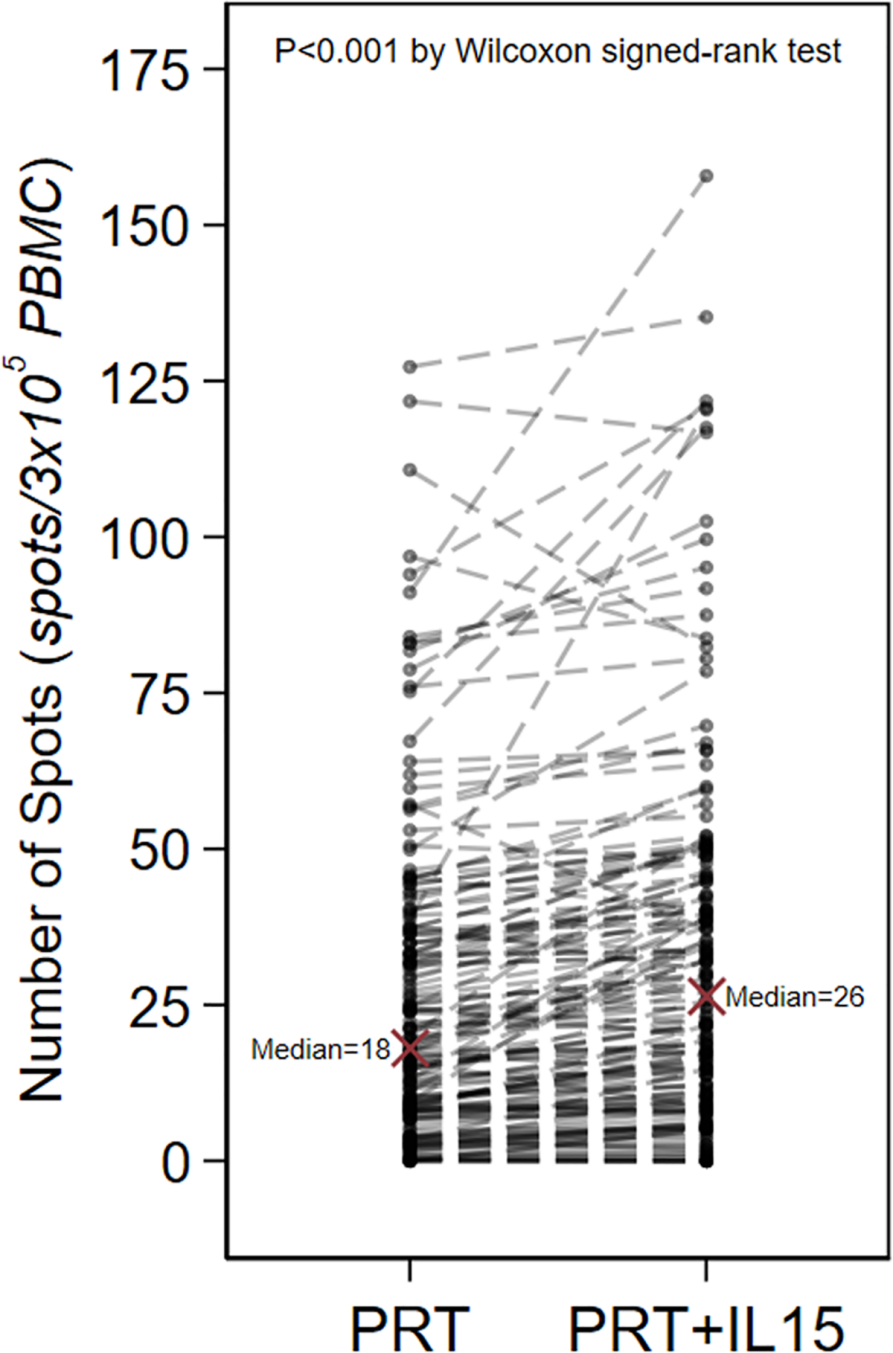
Median PRT and median PRT+IL5 (as number of spots) for each of the 168 patients. Dotted lines connect data belonging to the same patients. The number of spots was larger with median PRT+IL15 compared to median PRT (P<0.001 by Wilcoxon matched-pairs signed-ranks test).

Median d-sp ELISPOTs were correlated, to a lower extent, with PRT and PRT+IL15 (rho = 0.18, P = 0.021 and rho = 0.19, P = 0.016, respectively, **Fig 1A and 1B**), while PRT and PRT+IL15 were highly correlated with each other (rho = 0.96, P<0.001, **Fig 1C**). Median PRT values were significantly lower compared to median PRT+IL15 values (P<0.001; **Fig 2**). There was no statistically significant association between patients classified as d-sp ELISPOT positive and patients classified as PRT or PRT+IL15 positive (P = 0.64, and P = 0.44, respectively), while there was a strong association between positive PRT and positive PRT+IL15 patients (kappa coefficient of agreement: 0.66; P<0.001). The frequencies of PRT and PRT+IL15 spots did not differ between patients with a positive or negative d-sp ELISPOT (data not shown).

### Clinical outcomes of the study cohort

During the follow-up period, nine patients returned to dialysis and six died with a functioning graft (48-months graft survival: 90.2% [95%CI: 84.2 to 94.1]; 48-months death censored graft survival: 94.6% [89.8 to 97.1]. Three- and 48-month estimated eGFR were 51.1 and 45.6mL/min/1.73m^2^, respectively (48-month decrease in eGFR: -5.4mL/min/1.73m^2^ [95%CI: -7.9 to -3.0; P<0.001]. At 48 months after transplant, median proteinuria was 16 mg/24h and no patient had proteinuria above 0.5g/24h.

Fifty-seven (42.9%) and two (5.7%) of the deceased donor and living donor recipients respectively, developed DGF. Fifteen patients developed BPAR (8.9% [95%CI: 5.4 to 14.3]), including one case of antibody-mediated rejection. Among 131 patients with available Luminex SAB assessment after transplantation, *de novo* DSAs were detected in 17 cases (13%).

### Impact of pre-transplant d-sp ELISPOT and PRT assays on main graft outcomes

Donor-specific ELISPOT, PRT and PRT+IL15 were not associated with the main baseline clinical and epidemiological characteristics including number of previous transplants, type of ESRD, pre-transplant sensitization, donor and recipient age and gender, ethnicity, type of transplant or the number of HLA mismatches (**Table 2**).

**Table 2:** Association between recipients’ pre-transplant characteristics and pre-transplant number of spots of each assay.

	Difference in d-sp ELSPOT number of spots/3x10^5^PBMC (95%CI; P value)	Difference in median PRT number of spots/3x10^5^PBMC (95%CI; P value)	Difference in median PRT+IL15 number of spots/3x10^5^PBMC (95%CI; P value)
**Age≥60yrs vs <60yrs**	+2 (-13 to +17; P = 0.79)	+2 (-5 to +10; P = 0.55)	+2 (-7 to +11; P = 0.69)
**Male vs female**	+2 (-15 to +18; P = 0.84)	+1 (-7 to +10; P = 0.75)	+1 (-9 to +11; P = 0.84)
**Dialysis vintage≥5yrs vs <5yrs**	-11 (-34 to +13; P = 0.37)	+1 (-12 to +14; P = 0.92)	+2 (-13 to +18; P = 0.77)
Glomerulonephritis vs other primary renal diseases	+8 (-11 to +27; P = 0.41)	+5 (-6 to +16; P = 0.36)	+6 (-7 to +19; P = 0.37)
**Living vs deceased donor**	-5 (-22 to +13; P = 0.60)	+2 (-7 to +11; P = 0.61)	+6 (-6 to +18; P = 0.34)
**Re-transplantation vs first transplant**	-8 (-34 to +18; P = 0.55)	+1 (-14 to +16; P = 0.88)	-3 (-18 to +11; P = 0.64)
**cPRA > 5% per <5%**	-1 (-18 to +16; P = 0.89)	-1 (-18 to +16; P = 0.89)	-7 (-20 to +7; P = 0.34)
**Thymoglobulin**	-14 (-29 to +1; P = 0.073)	-2 (-11 to +8; P = 0.75)	-2 (-12 to +8; P = 0.69)
**Cyclosporine vs tacrolimus**	-13 (-33 to +8; P = 0.22)	+3 (-11 to +16; P = 0.72)	+4 (-11 to +20; P = 0.56)
**Steroid withdrawal**	-26 (-41 to -11; P = 0.001)	+2 (-6 to +10; P = 0.62)	+6 (-3 to +14; P = 0.21)

Difference (95 percent confidence interval and P vale) of the number of spots of each pre-transplant assay between dichotomous categories defined by pre-transplant recipients’ characteristics. 95%CI, 95 percent confidence interval; cPRA, calculated Panel-Rective Antibody. Median PRT and median PRT+IL15, median number of spots against the six B-cell lines.

Unexpectedly, patients who underwent steroid withdrawal had significantly lower pre-transplant d-sp ELISPOTs compared to patients maintained on steroids (**Table 2**). There was no association between pre-transplant d-sp ELISPOT, PRT or PRT+IL15 and DGF (data not shown).

**Fig 3:**
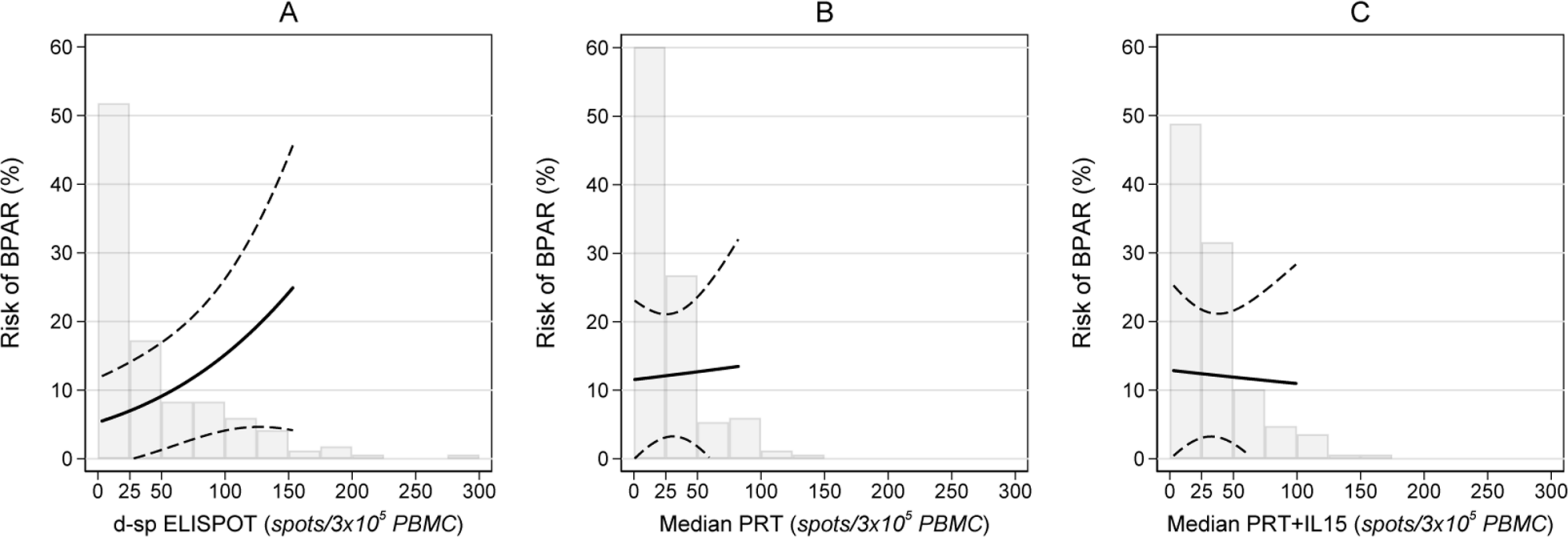
**Risk of BPAR (i.e. proportion of patients developing BPAR) according to the number of IFN-γ spots for d-sp ELISPOT (A), median PRT (B), and median PRT+IL15 (C).** The solid line represents the risk of BPAR, the dashed lines represent the upper and lower 95% confidence intervals. The risk of BPAR significantly increased with the number of spots of d-sp ELISPOT (P = 0.042), whereas it did not increase with the number of spots of Median PRT and Median PRT+IL15 (P = 0.87 and P = 0.88, respectively). The superimposed histograms report the frequency distribution of the data values in the study population. The spread of the data values was larger with d-sp ELISPOT compared to Median PRT and Median PRT+IL15. Outside the range of the actual data distribution the risk of BPAR is not reported because it would otherwise represent an inaccurate extrapolation of the BPAR risk estimates. The plotted risk of BPAR is adjusted for recipient and donor age, living (vs deceased) donor, cold ischemia time, Thymoglobulin induction, re-transplantation, pre-transplant HLA antibodies, HLA A/B AND HLA DR mismatch, glomerulonephritis as primary renal disease, dialysis vintage, and prednisone withdrawal (*i*.*e*., Model 2 in Table 3). Every covariate was set to the mean value in the study population, the indicator variate prednisone withdrawal was set to zero (*i*.*e*., no withdrawal).

Donor-specific ELISPOT positivity was associated with a significantly higher risk of BPAR (12/81[15%] vs. 3/87 [3%] for d-sp ELISPOT positive vs. negative, P = 0.013 respectively; adjusted Odds Ratio, aOR: 4.20 [95%CI: 1.06 to 21.73; P = 0.041], **Table 3**). A positive pre-transplant d-sp ELISPOT predicted BPAR with a negative and positive predictive value of 96% (95%CI: 94 to 99%), and 15% (95%CI: 9 to 20%), respectively. Conversely, there was no association between positive PRT or PRT+IL15 and BPAR (BPAR risk in positive vs negative patients: 6/71 [8%] vs 9/97 [9%], P = 0.54; and 6/81 [7%] vs 9/87 [10%], P = 0.59 for PRT and PRT+IL15, respectively). When expressed per one standard deviation unit increase in the number of spots, the aOR of BPAR was 1.79 (95%CI: 1.02 to 3.10: P = 0.042) for d-sp ELISPOT, and 1.06 (0.50 to 1.95; P = 0.87) and 0.95 (0.42 to 1.82; P = 0.88) for median PRT and median PRT+IL15, respectively (**Table 3**). **Fig 3** shows the adjusted predicted risk of BPAR (i.e. proportion of patients developing BPAR) according to the number of spots of d-sp ELISPOT, median PRT, and median PRT+IL15, with superimposed the histograms of the actual data distribution of the assay test results in the study population. Increased d-sp ELISPOT spot frequencies were associated with four-time increased risk of BPAR (from zero to 150 spots BPAR risk increased from approximately 5 to 20%), while increased median PRT and median PRT+IL15 spots were not associated with increased BPAR risk.

**Table 3:** Crude and adjusted odds ratio of BPAR associated with d-sp ELISPOT.

	Crude Analysis OR (95% CI; P value)	Adjusted Analysis, Model 1 OR (95% CI; P value)	Adjusted Analysis, Model 2 OR (95% CI; P value)
Positive vs negative d-sp ELISPOT			
Whole population	4.87 (1.48–21.99; P = 0.008)	3.70 (1.02–17.84; P = 0.046)	4.20 (1.06–21.73; P = 0.041)
Not receiving Thymoglobulin	6.56 (1.69–43.36; P = 0.005)	5.32 (1.25–37.01; P = 0.022)	7.87 (1.52–68.88; P = 0.012)
Number of d-sp ELISPOTs			
Whole population	1.91 (1.25–2.99; P = 0.004)	1.75 (1.08–2.84; P = 0.024)	1.79 (1.02–3.10; P = 0.042)
Not receiving Thymoglobulin	1.95 (1.24–3.17; P = 0.004)	1.81 (1.09–3.06; P = 0.022)	1.89 (1.07–3.37; P = 0.029)

OR, Odds, Ratio; 95%CI, 95 percent Confidence Interval; BPAR, Biopsy-prove acute rejection; d-sp ELISPOT, donor-specific ELISPOT

The Odds Ratio associated to the number of d-sp ELISPOT is expressed per one standard deviation unit increase (i.e. 50 spots/3x10^5^ PBMC)

Model 1, adjusted for recipient and donor age, Thymoglobulin induction (whole population only), and prednisone withdrawal

Model 2, adjusted for recipient and donor age, living (vs deceased) donor, cold ischemia time, Thymoglobulin induction (whole population only), re-transplantation, pre-transplant HLA antibodies, HLA A/B AND HLA DR mismatch, glomerulonephritis as primary renal disease, dialysis vintage, and prednisone withdrawal.

The association between d-sp ELISPOT and BPAR became stronger after excluding the 41 patients who received thymoglobulin induction. In patients with positive d-sp ELISPOT not induced with thymoglobulin, the incidence of BPAR was 12/66 (18%) vs 2/61 (3%) in patients with negative d-sp ELISPOT (P = 0.009; aOR 7.97 [1.52 to 68.88; P = 0.012], **Table 3**). When expressed per one standard deviation unit increase in the number of spots, the aOR of BPAR increased to 1.89 (1.07–3.37; P = 0.029) for d-sp ELISPOT (**Table 3**), whereas the aOR remained unchanged for median PRT and for median PRT+IL15 (aOR 1.12 [0.51–2.23; P = 0.75], and 1.02 [0.44–2.06; P = 0.96], respectively).

There was no association between pre-transplant d-sp ELISPOT, PRT, or PRT+IL15 and *de novo* DSA in the subset of 131 patients evaluated (*de novo* DSA risk in positive vs negative patients: 6/70 [8.6%] vs 11/61 [18%], P = 0.11; 5/58 [8.6%] vs 12/73 [16.4%], P = 0.19; 7/66 [10.6%] vs 10/65 [15.4%], P = 0.42 for donor-reactive d-sp ELISPOT, PRT, or PRT+IL15, respectively). Similarly, the analysis based on spots considered as a continuous variable yielded non-significant findings (data not shown).

### Graft function progression and survival

**Fig 4:**
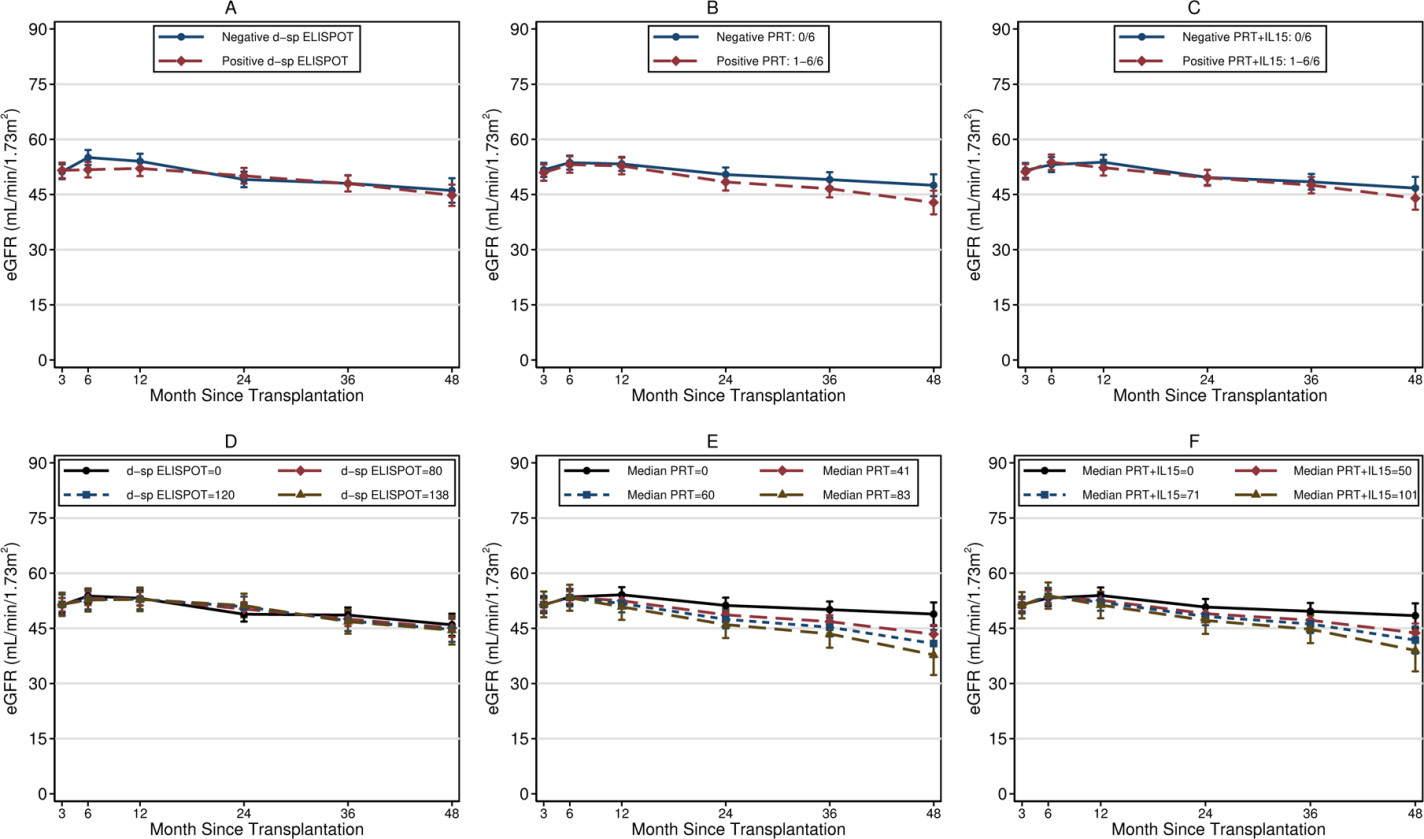
**Fitted means of 48-months eGFR decline from multiple regression models for repeated measures (see text) in patients with positive and negative d-sp ELISPOT (A), positive and negative PRT (B), positive and negative PRT+IL15 (C), and in hypothetical patients having number of IFN-γ spots equal to the 0°, 80°, 90°, and 95° centile of the study population for d-sp ELISPOT (D), for median PRT (E), and for median PRT+IL15 (F).** The 48-months eGFR decline did not differ significantly when comparing positive vs negative assays, but it did differ when examining the relation with the numerical variable number-of-spots of median PRT (**E**) and of median PRT+IL15 (**F**). According to multiple regression models, the 48-months eGFR declined by -3.4mL/min/1.73m^2^ (95%CI: -5.8 to -1.1; P = 0.005) and by -2.8 mL/min/1.73m^2^ (-5.2 to -0.3; P = 0.037) per one standard deviation unit increase in the number of IFN-γ spots of median PRT and median PRT+IL15, respectively. Panels **D**, **E**, and **F** report the fitted 48-months eGFR decline of hypothetical patients having number of IFN-γ spots equal to the 0°, 80°, 90°, and 95° centile of the study population to provide a visual appraisal of the fitted relation mentioned above between the number of IFN-γ spots and eGFR decline. Dots represent predicted means from the fitted multiple regression models for repeated measures, vertical bars represent 95% confidence intervals. Regression models were adjusted for 3-month eGFR, recipient and donor age, living (vs deceased) donor, cold ischemia time, Thymoglobulin induction, re-transplantation, pre-transplant HLA antibodies, HLA A/B AND HLA DR mismatch, glomerulonephritis as primary renal disease, dialysis vintage, and prednisone withdrawal.

After adjusting for 3-month eGFR and for baseline clinical and demographic characteristics, median PRT and median PRT+IL15, but not d-sp ELISPOT, were significantly associated with a sharper decline of 3-48months eGFR (**Fig 4**). The eGFR decline increased by -3.4mL/min/1.73m^2^ (95%CI: -5.8 to -1.1; P = 0.005) and by -2.8 mL/min/1.73m^2^ (-5.2 to -0.3; P = 0.037) per one standard deviation unit increase in the number of spots for median PRT and for median PRT+IL15, respectively (**Fig 4**). However, positive PRT and PRT+IL15 were not significantly correlated with 48-month eGFR decline (difference in 48 months eGFR decline between positive and negative patients: -3.9 mL/min/1.73m^2^ [-8.5 to +0.7; P = 0.096] and -2.3 [-7.0 to +2.3; P = 0.32] for PRT and PRT+IL15, respectively) (**Fig 4**). There was no relationship between any of the assays studied and 24h-proteinuria at 48 months after transplant (data not shown).

Being positive or negative for both the d-sp ELISPOT and the PRT (- or + IL15) assay did not provide any prognostic advantage in predicting BPAR or eGFR change beyond every single assay used alone (data not shown). Crude and adjusted analyses showed no significant association between and any of the assays studied and uncensored or death-censored graft failure (data not shown).

## Discussion

Our large study of deceased and living kidney transplant recipients shows that positive d-sp ELISPOT, but not PRT, identifies patients at increased risk of BPAR after transplantation, whereas patients with high pre-transplant PRT display significant graft function loss over a 4-year follow-up period. Addition of IL15 did not increase the predictive power of the PRT assay.

Previous studies testing the predictive power value of d-sp ELISPOT or PRT in kidney transplant recipients prior to transplantation largely included small cohorts, have reported a disparity of results and did not formally compare the two assays, while our data from a large series of transplanted individuals allowed to assess the potential complementary characteristics of both assays.

Recent works have shown a strong association between positive pre-transplant d-sp ELISPOT and increased risk of BPAR after transplantation, particularly TCMR, and especially, in patients not receiving T-cell depleting induction therapies [8,14,18]. Faddoul G and colleagues [33] reported a close association between d-sp alloreactivity evaluated with d-sp ELISPOT and an increased risk of BPAR and worse graft function at 2 years in positive patients. Our current findings confirm and expand previous evidence in a larger cohort of patients, where the relationship between pre-transplant d-sp ELISPOT and a higher risk of BPAR was mainly driven by patients not induced with Thymoglobulin. Additionally, in our group of patients, high PRT responses also associated with worse allograft function progression. Altogether suggesting that preformed T cell alloreactivity main negatively impact post-transplant graft outcome.

Despite the statistically significant association between pre-transplant d-sp ELISPOT and higher risk of BPAR, only 15% of patients with positive test developed the event. On the other hand, amongst subjects with negative assay, virtually no one developed BPAR (only 3%) thus, highlighting the high negative predictive value of the assay enabling an accurate capacity to identify those patients at low immunological risk that could eventually benefit of receiving less immunosuppression.

We did not find any association between a positive pre-transplant d-sp ELISPOT and worse allograft function progression after transplantation. Nonetheless, while some studies have showed such correlation, particularly among patients not receiving T-cell induction therapy [14,20], some other groups have not been able to find such association before transplantation [8,13,16,34,35] but have conversely found a consistent relationship between worse kidney allograft function progression and the d-sp ELISPOT when assessed after transplantation suggesting a much close illustration of the on-going anti-donor T-cell alloimmune response of transplant patients.

To the best of our knowledge, the current study is the largest work testing the association between pre-transplant PRT and graft outcomes. In contrast with previous few smaller studies [22], we did not detect a relationship between PRT and BPAR. A previous study identified an inverse nonsignificant trend between pre-transplant PRT and eGFR. The large sample size of our cohort and long-term follow-up, allowed us to find a significant association between the frequency of pre-transplant PRT and PRT+IL15 and eGFR decline at 4 years. This association suggests that patients with a broad pre-transplant alloreactive T-cell repertoire, expressed by means of high PRT, may be at increased risk of the formation of crossreactive T and B cells with allo- and auto-reactive specificities that may lead to progressive but smoldering subclinical allograft damage. Unfortunately, lack of surveillance graft biopsies or measurement of circulating auto- or allo-antibodies in a significant fraction of patients prevented us from confirming this hypothesis.

Adding IL15 to the PRT did not increase the predictive power of the assay, indicating that the circulating CD8^+^CD28^-^ Tmem measured before engraftment do not play a major role in the pathogenesis of subsequent allograft injury, at least in patients on calcineurin inhibitor-based immunosuppression. This is consistent with our preliminary data suggesting that the number of these cells before transplant predicted acute rejection only in kidney transplant recipients receiving costimulation blockade-based immunosuppression with CTLA4Ig (Cravedi P, Gandolfini I, Donadei C, et al. Pre-Transplant Panel Reactive T Cells (PRT) with IL-15 as a Risk-Stratifier of Acute Rejection in Kidney Transplant Patients on Belatacept Therapy. American Transplant Congress. Chicago, May 2017. Abstract). Further studies, however, are needed to define the utility of PRT+IL15 in predicting graft outcomes in kidney transplant recipients.

We found no relationship between any of the pre-transplant assays and the development of DSA. While missing data on DSA development may have prevented us from detecting such relationship, these data are in agreement with our 2017 recent study [8] showing that only post-transplant, but not pre-transplant d-sp ELISPOT can inform on the risk of developing DSA. This data together with the absence of association with graft function progression over time highly suggest that monitoring anti-donor T-cell alloreactivity after kidney transplantation may be particularly useful to gain more insight about the alloimmune state of transplant patients after having received the initial high burden of induction immuosuppression and thus, more accurately indicate how such immune state may progressively impact on long-term allograft outcomes.

We acknowledge that our study has some limitations. Firstly, it was a single-center, retrospective study. However, main aim of this study was to perform the first large comparison between the two main assays to measure T-cell alloreactivity that could be implemented in clinical practice in the short term. In addition, the use of only six B-cell lines might have restricted the broad allogenic repertoire of the transplant recipients evaluated in this study and therefore, might have prevented a more granular differentiation between patients, as it occurs with the PRA assays. However, the findings of the current study set the basis for subsequent investigations to test the predictive power of PRT based on a more extensive panel of B-cell lines.

Due to the limited amount of PBMC available, we could not perform d-sp ELISPOT in the presence of IL15 and thus, future studies will be important in assessing are warranted to test the utility of such assay in predicting graft outcomes.

## Conclusions

Our findings confirm and further expand previous evidence showing that measuring alloreactive Tmem before transplantation by d-sp ELISPOT or PRT allows to predict relevant immune-mediated transplant outcomes that would not be forecasted by current standard clinical and immunological evaluations. Present findings do not support the use of IL15 in the PRT assay, but further studies are needed to define its utility, especially in patients not receiving calcineurin inhibitor immunosuppression. Our data set the basis of prospective studies formally testing the hypothesis that tailoring immunosuppression based on the joint use of d-sp ELISPOT and PRT improves patients’ outcomes.

## Supporting information

S1 Fig Analysis of the best choice among different combinations of PBMCs and B cells in order to reproduce the results from the standard PRT assay, which is traditionally based on a 300.000 PBMCs to 100.000 B cells ratio per well. Each plot represents the linear correlation between a PBMCs to B cells ratio combination (x-axis) selected in order to minimize the number of cell used, and the standard 300.000 PBMCs/100.000 B cells combination (y axis). The 100.000 PBMCs to 60.000 B cells ratio (left-lower-most panel) showed an excellent correlation with the standard 300.000 PBMCs to 100.000 B cells ratio (R2 = 0.94) and was therefore used for the PRT assays in the current study. Each dot represents the average of two wells.
(DOCX)

S2 Fig Percentage of expression of HLA A, B and DR alleles and coverage to donor HLA repertoires provided by B cell lines. Expression of B cell panel HLA alleles represented 61.95%, 59.27% and 78.86% of expression over all HLA A, HLA B and HLA DR alleles expressed, respectively. Red bars represent HLA alelles that were in common with the B cell lines.
(DOCX)

S1 Table HLA typing of the six B cell lines used as stimulators in the PRT assay. (DOCX)
