# Supplementary material for: Impact of preformed T-cell alloreactivity by means of donor-specific and panel of reactive T cells (PRT) ELISPOT in kidney transplantation
Source: PLoS One. 2018 Jul 30;13(7):e0200696. doi: 10.1371/journal.pone.0200696 (PMC6066206; doi:10.1371/journal.pone.0200696)
Supplement: S1 Fig — Each plot represents the linear correlation between a PBMCs to B cells ratio combination (x-axis) selected in order to minimize the number of cell used, and the standard 300.000 PBMCs/100.000 B cells combination (y axis). The 100.000 PBMCs to 60.000 B cells ratio (left-lower-most panel) showed an excellent correlation with the standard 300.000 PBMCs to 100.000 B cells ratio (R2 = 0.94) and was therefore used for the PRT assays in the current study. Each dot represents the average of two wells. (DOCX) [file pone.0200696.s001.docx]

**S1 Fig**. Analysis of the best choice among different combinations of PBMCs and B cells in order to reproduce the results from the standard PRT assay, which is traditionally based on a 300.000 PBMCs to 100.000 B cells ratio per well. Each plot represents the linear correlation between a PBMCs to B cells ratio combination (x-axis) selected in order to minimize the number of cell used, and the standard 300.000 PBMCs/100.000 B cells combination (*y* axis). The 100.000 PBMCs to 60.000 B cells ratio (left-lower-most panel) showed an excellent correlation with the standard 300.000 PBMCs to 100.000 B cells ratio (R2=0.94) and was therefore used for the PRT assays in the current study. Each dot represents the average of two wells.
