# Supplementary material for: Impact of preformed T-cell alloreactivity by means of donor-specific and panel of reactive T cells (PRT) ELISPOT in kidney transplantation
Source: PLoS One. 2018 Jul 30;13(7):e0200696. doi: 10.1371/journal.pone.0200696 (PMC6066206; doi:10.1371/journal.pone.0200696)
Supplement: S2 Fig — Expression of B cell panel HLA alleles represented 61.95%, 59.27% and 78.86% of expression over all HLA A, HLA B and HLA DR alleles expressed, respectively. Red bars represent HLA alelles that were in common with the B cell lines. (DOCX) [file pone.0200696.s002.docx]

**S2 Fig**. Percentage of expression of HLA A, B and DR alleles and coverage to donor HLA repertoires provided by B cell lines. Expression of B cell panel HLA alleles represented 61.95%, 59.27% and 78.86% of expression over all HLA A, HLA B and HLA DR alleles expressed, respectively. Red bars represent HLA alelles that were in common with the B cell lines.
