# Supplementary material for: Impact of preformed T-cell alloreactivity by means of donor-specific and panel of reactive T cells (PRT) ELISPOT in kidney transplantation
Source: PLoS One. 2018 Jul 30;13(7):e0200696. doi: 10.1371/journal.pone.0200696 (PMC6066206; doi:10.1371/journal.pone.0200696)
Supplement: S1 Table — (DOCX) [file pone.0200696.s003.docx]

**S1 Table. HLA typing of the six B cell lines used as stimulators in the PRT assay.**

|  | | | | | | | | | | | | | | |
| --- | --- | --- | --- | --- | --- | --- | --- | --- | --- | --- | --- | --- | --- | --- |
| B cell line | **A-1** | **A-2** | **B-1** | **B-2** | **C-1** | **C-2** | **DRB1-1** | **DRB1-2** | **DRB3** | **DQA1-1** | **DQA1-2** | **DQB1-1** | **DQB1-2** |  |
| B1 | A*30 | A*34 | B*15 (72) | B*44 | C*04 | C*04 | DRB1*11 | DRB1*15:03 | DRB3*02 | DQA1*01 | DQA1*05 | DQB1*03:19(7) | DQB1*06 |  |
| B2 | A*03 | A*03 | B*44 | B*44 | C*05 | C*16 | DRB1*04 | DRB1*11 | DRB3*02 | DQA1*03:01 | DQA1*05 | DQB1*03 (7) | DQB1*03(8) |  |
| B3 | A*02 | A*30 | B*18 | B*42 | C*05 | C*17 | DRB1*03 (17) | DRB1*15:03 | DRB3*02 | DQA1*01 | DQA1*05:01 | DQB1*02 | DQB1*06 |  |
| B4 | A*03 | A*24 | B*07 | B*35 | C*04 | C*07 | DRB1*07 | DRB1*13 | DRB3*03:01 | DQA1*01 | DQA1*02:01 | DQB1*03(9) | DQB1*06 |  |
| B5 | A*11 | A*26 | B*15 (62) | B*51 | C*04 | C*14 | DRB1*03 (17) | DRB1*11 | DRB3*02 | DQA1*05 | DQA1*05 | DQB1*02 | DQB1*03(7) |  |
| B6 | A*03 | A*30 | B*13 | B*38 | C*06 | C*12 | DRB1*07 | DRB1*13 | DRB3*01 | DQA1*01 | DQA1*02:01 | DQB1*02 | DQB1*06 |  |
| HLA typing of the 6 B cell lines used as stimulators in the PRT+/- IL15 assay. Each allele is reported in a separate column. | | | | | | | | | | | | | |  |
